# Selective elimination of cancer cells by the adenovirus E4orf4 protein in a *Drosophila* cancer model: a new paradigm for cancer therapy

**DOI:** 10.1038/s41419-019-1680-4

**Published:** 2019-06-11

**Authors:** Helit Rosen, Rakefet Sharf, Antonina Pechkovsky, Adi Salzberg, Tamar Kleinberger

**Affiliations:** 1 Department of Molecular Microbiology, The Rappaport Faculty of Medicine and Research Institute, Technion—Israel Institute of Technology, 3109601 Haifa, Israel; 2 Department of Genetics and Developmental Biology, The Rappaport Faculty of Medicine and Research Institute, Technion—Israel Institute of Technology, 3109601 Haifa, Israel

**Keywords:** Cancer models, Mechanisms of disease

## Abstract

The adenovirus (Ad) E4orf4 protein contributes to efficient progression of virus infection. When expressed alone E4orf4 induces p53- and caspase-independent cell-death, which is more effective in cancer cells than in normal cells in tissue culture. Cancer selectivity of E4orf4-induced cell-death may result from interference with various regulatory pathways that cancer cells are more dependent on, including DNA damage signaling and proliferation control. E4orf4 signaling is conserved in several organisms, including yeast, *Drosophila*, and mammalian cells, indicating that E4orf4-induced cell-death can be investigated in these model organisms. The *Drosophila* genetic model system has contributed significantly to the study of cancer and to identification of novel cancer therapeutics. Here, we used the fly model to investigate the ability of E4orf4 to eliminate cancer tissues in a whole organism with minimal damage to normal tissues. We show that E4orf4 dramatically inhibited tumorigenesis and rescued survival of flies carrying a variety of tumors, including highly aggressive and metastatic tumors in the fly brain and eye discs. Moreover, E4orf4 rescued the morphology of adult eyes containing *scrib*^*−*^ cancer clones even when expressed at a much later stage than *scrib* elimination. The E4orf4 partner protein phosphatase 2A (PP2A) was required for inhibition of tumorigenesis by E4orf4 in the system described here, whereas another E4orf4 partner, Src kinase, provided only minimal contribution to this process. Our results suggest that E4orf4 is an effective anticancer agent and reveal a promising potential for E4orf4-based cancer treatments.

## Introduction

The adenovirus E4orf4 protein is a multifunctional viral regulator, which contributes to regulation of the progression of viral infection^1,2^ and to inhibition of the cellular DNA damage response (DDR), thus increasing the efficiency of adenovirus replication^3,4^. When expressed alone E4orf4 induces a p53- and caspase-independent mode of cell-death, which can lead to classical caspase-dependent apoptosis in some cell lines^5–8^. E4orf4 cell-death signaling is conserved in several organisms, including yeast, *Drosophila*, and mammalian cells^9–12^. Our studies in normal *Drosophila* tissues demonstrated that E4orf4 induced both caspase-dependent and –independent cell-death in the fly, but also inhibited classical apoptosis, thereby causing minimal tissue damage and a marginal effect on fly survival^12^. Studies in mammalian cells revealed that E4orf4-induced cell-death was more efficient in oncogene-transformed cells than in normal cells^13^, indicating that investigation of E4orf4 signaling may have practical implications for cancer therapy. The cancer selectivity of E4orf4-induced cell-death may result from a combination of several E4orf4 activities that interfere with various pathways of cell regulation^1,2^.

Several E4orf4 cellular partners that contribute to E4orf4-induced cell-death have been described, including protein phosphatase 2A (PP2A) and Src kinases^2^. E4orf4 binds the heterotrimeric PP2A holoenzyme through direct association with its regulatory B55 subunit^14,15^.

The model organism *Drosophila melanogaster* has contributed considerably to the study of cancer and to identification of novel cancer therapeutics^16–21^. Various mutations are known to cause tumorigenesis in *Drosophila* including those affecting tumor suppressor genes that are required for normal cell polarity and asymmetric cell divisions, such as *scribble* (*scrib*)^22^. Human homologs of these genes are associated with the formation of diverse types of cancers^23,24^. When *scrib* mutant cells are surrounded by similar cells they develop tumors, but when surrounded by normal cells they do not^25^, unless supplemented by constitutive activation of the Ras pathway, which confers a proliferation advantage and leads to formation of aggressive, metastatic tumors^25,26^. Activated Ras (*Ras*^*V12*^) also cooperates with activation of the phosphoinositide 3-kinase (PI3K) pathway to generate more aggressive tumors^27^.

Here, we show that E4orf4 expression dramatically inhibited the development of various aggressive tumors without greatly affecting healthy tissues, and could restore normal tissue morphology even when expressed at a late stage. The role of E4orf4 partners in this process is also described.

## Results

### E4orf4 counteracts *Ras*^*V12*^-induced impairment of tissue differentiation and fly survival in a dose-dependent manner

**Fig. 1:**
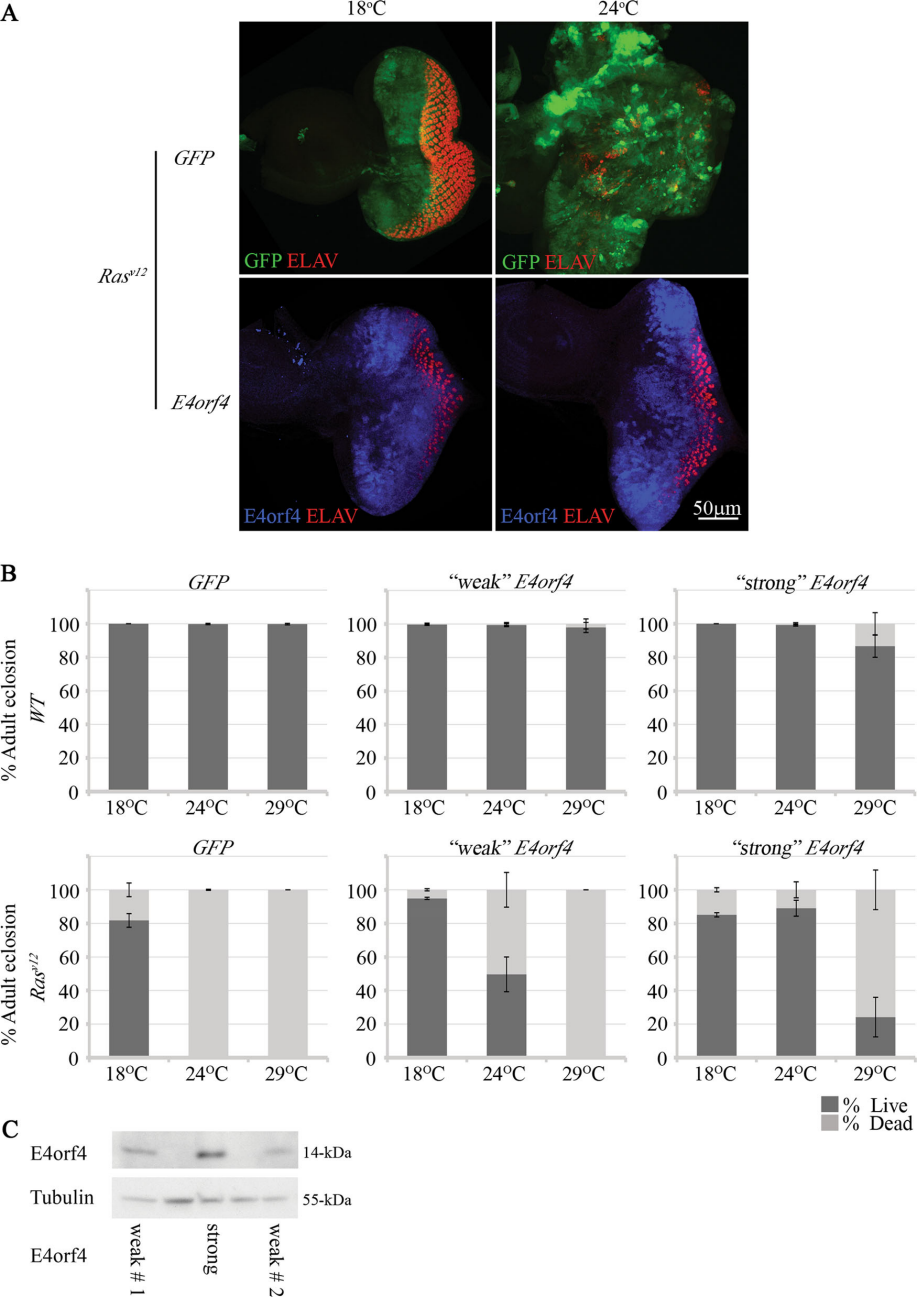
E4orf4 counteracts *Ras*^*V12*^-induced impairment of tissue differentiation and fly survival in a dose-dependent manner. **a** Eye-antennal imaginal discs expressing *Ras*^*V12*^ under the regulation of *ey-GAL4* together with *GFP (ey-GAL4* *>* *UAS-Ras*^*V12*^*; FRT82B*, *UAS-GFP)* or E4orf4 *(ey-GAL4* *>* *UAS-Ras*^*V12*^*; FRT82B*, *UAS-E4orf4-17*.*22)* are shown. Discs were dissected from age-matched 3rd-instar larvae grown at 18 ^o^C or 24 ^o^C. The discs were stained with antibodies to E4orf4 (blue) and ELAV (red) and were analyzed by confocal microscopy. All images were taken at the same magnification (x40) and represent projections of multiple sections. A representative eye disc is shown in each picture. The 50 μm scale bar applies to all the pictures. It should be noted that age-matched larvae were utilized, but E4orf4-expressing larvae were consistently slower to differentiate. **b** Percentage of adult eclosion (“Live”) and of non-eclosed pupae (“Dead”) was determined in flies expressing *GFP* (labeled as *WT)* or *Ras*^*V12*^ under the regulation of *ey-GAL4* together with another copy of *GFP* (*ey-GAL4* *>* *UAS-GFP;FRT82B*, *UAS-GFP*, and *ey-GAL4* *>* *UAS-Ras*^*V12*^*;FRT82B*, *UAS-GFP)* or E4orf4. Two different fly strains harboring an E4orf4 transgene were tested. These strains express the viral protein to different levels: “strong”: *UAS-E4orf4-17*.*22* (*ey-GAL4* *>* *UAS-GFP;FRT82B*,*UAS-E4orf4-17*.*22* and *ey-GAL4* *>* *UAS-Ras*^*V12*^*;FRT82B*,*UAS-E4orf4-17*.*22)* and “weak”: *UAS-E4orf4-attP2*, also called “weak1” in **c** below *(ey-GAL4* *>* *UAS-GFP;UAS-E4orf4-attP2* and *ey-GAL4* *>* *UAS-Ras*^*V12*^*;UAS-E4orf4-attP2)*. The flies were grown at the indicated temperatures. *N* > 300, collected from three independent experiments. **c** Protein extracts were prepared from ten 3rd instar larvae expressing “strong” E4orf4 and two types of “weak” E4orf4 under the regulation of *ey-GAL4* (*ey-GAL4* *>* *UAS-E4orf4-17*.*22*, *ey-GAL4* *>* *UAS-E4orf4-attP2* (weak#1), and *ey-GAL4* *>* *UAS-E4orf4-attP40* (weak #2)). A representative western blot is shown, stained with antibodies to E4orf4 and to Tubulin, which served as a loading control

The consequences of *Ras*^*V12*^ expression in the presence or absence of E4orf4 were initially examined in eye imaginal discs. *Ras*^*V12*^ was expressed together with either E4orf4 or a control *GFP* gene using the *UAS-GAL4* system driven by the *eyeless* (*ey*) promoter, which drives expression in the eye disc, as well as in regions of the brain^28,29^. *GAL4*-driven expression rises with increasing temperatures, facilitating investigation of dose-dependent effects. As seen in Fig. 1a, the expression of *Ras*^*V12*^ together with *GFP* at 24 ^o^C resulted in loss of normal eye disc morphology and a significant increase in disc size indicating induction of uncontrolled proliferation and differentiation defects. The differentiation defects were also visualized by the unorganized staining of the neuronal differentiation marker ELAV. The expression of E4orf4 counteracted the *Ras*^*V12*^-induced over-proliferation of cells and the loss of normal differentiation as evident by the smaller size and normal shape of the E4orf4 *Ras*^*V12*^ expressing discs.

In the experiments described in Fig. 1a, we noticed a reduced proportion of adult eclosion upon *Ras*^*V12*^ expression at high temperatures, possibly resulting from *ey*-driven *Ras*^*V12*^ effects in the brain. We therefore examined the ability of E4orf4 to rescue the *Ras*^*V12*^-induced lethality at various temperatures (Fig. 1b). Two different *UAS*-*E4orf4* fly strains were utilized, one expressing higher E4orf4 levels than the other when driven by *ey-GAL4* (Fig. 1c). In a *WT* background (*ey* *>* *GFP*), even high levels of E4orf4 (“strong”) had an insignificant effect on adult fly viability at 18 ^o^C and 24 ^o^C (*p* > 0.1) and reduced viability by 13% at most at 29 ^o^C (*p* = 0.013). These results are consistent with our previous findings showing that expression of E4orf4 in normal fly tissues caused only mild effects^12^. In contrast, strong E4orf4 expression rescued the 100% lethality induced by *ey* *>* *Ras*^*V12*^ at 24 ^o^C and 29 ^o^C to 89% and 24% viability, respectively (*p* < 0.012). Weaker E4orf4 expression led to rescue of 50% viability at 24 ^o^C (*p* < 0.0001), but could not rescue the *Ras*^*V12*^-induced lethality at 29 ^o^C. These findings confirm that the dose-dependence of E4orf4 activity, which was previously reported in *WT* flies^12^ is also sustained in a *Ras*^*V12*^ background, and reveal that E4orf4 counteracts *Ras*^*V12*^-induced lethality.

### E4orf4 inhibits the formation of highly aggressive tumors in the fly

The preliminary experiments described in Fig. 1 investigated *Ras*^*V12*^ and E4orf4 effects occurring when these genes were expressed broadly in the developing eye. However, since tumor growth is significantly influenced by the microenvironment, additional investigation of E4orf4 effects on tumorigenesis was conducted in clonally induced tumors. For this purpose, the mosaic analysis with a repressible cell marker (MARCM) method was utilized as described previously^26^ to induce mitotic GFP-labeled clones in the eye discs and brain. The overexpression of *Ras*^*V12*^ induces non-invasive tumors in flies^26,30^. Since we wished to examine the ability of E4orf4 to eliminate various types of tumors, including highly aggressive, metastatic tumors, we analyzed mitotic clones carrying genetic alterations including *Ras*^*V12*^ activation and/or inactivation of the tumor suppressor *scribbled* (*scrib*), a regulator of cell polarity^23^. The clones were produced by *ey*-driven *FLP* recombinase, which mediated recombination between chromosome arms, leading to homozygous loss of *scrib* and to loss of the *UAS-GAL4* system inhibitor *Gal80*, facilitating *GAL4*-driven expression of *UAS*-regulated genes within the clones^26^. Flies generated using this system carried one of four types of mitotic clones, including *WT* clones, *scrib*^*−*^ clones, clones expressing *Ras*^*V12*^, and *scrib*^*−*^ clones that expressed *Ras*^*V12*^. Each of these clones also expressed either the strong E4orf4 transgene or a control *RFP* gene.

**Fig. 2:**
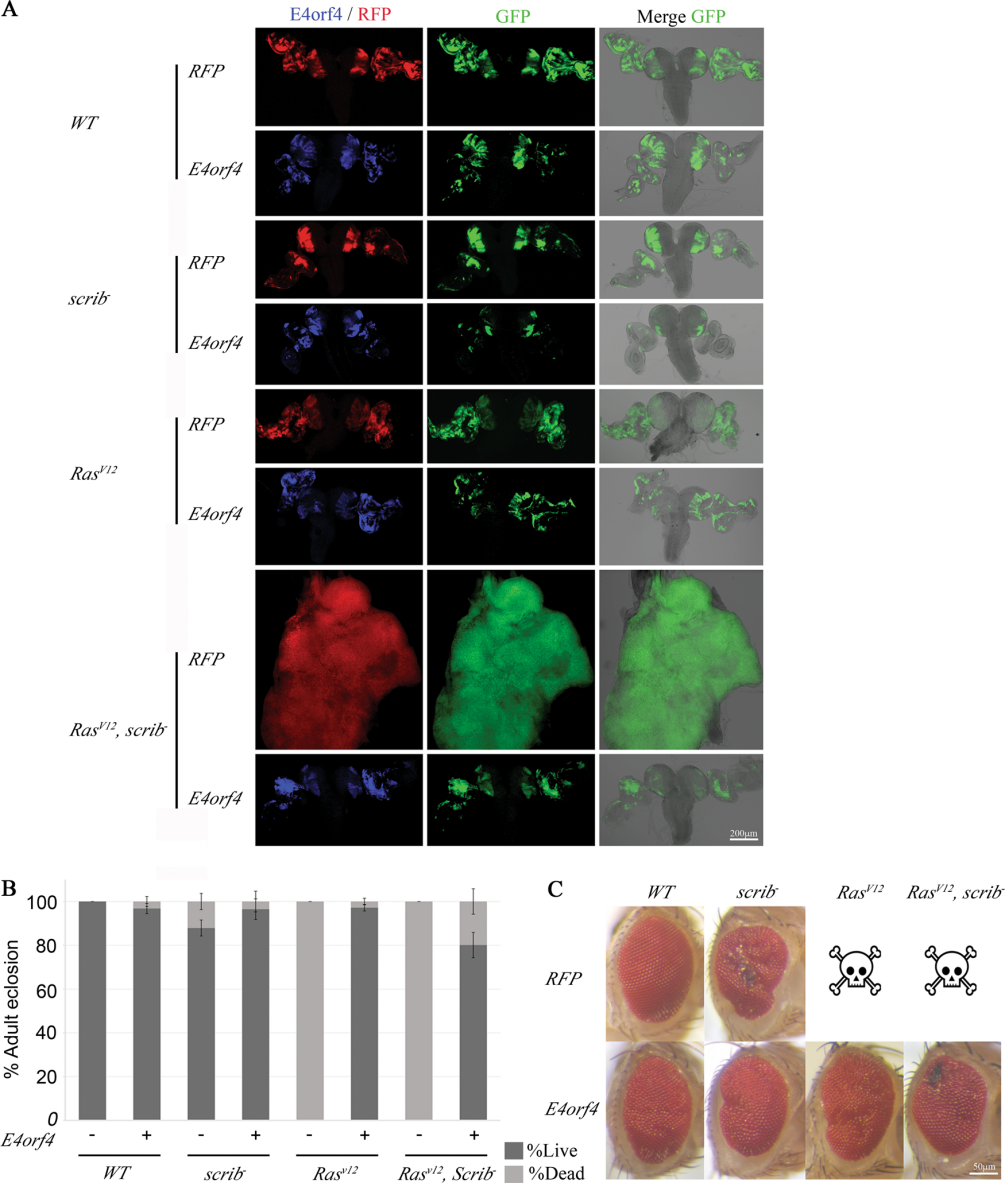
E4orf4 inhibits tumorigenesis in the fly. **a** Brains and eye-antennal discs harboring *ey-FLP*-induced clones were dissected from 3rd-instar larvae grown at 24 ^o^C and analyzed by confocal microscopy. *WT* and mutant clones of the indicated genotypes are marked by GFP fluorescence (middle column); the presence of E4orf4 (blue) or control RFP (red) was detected by immunostaining or RFP fluorescence (left column). The morphology of brains and eye-antennal discs is shown in the right column together with GFP fluorescence to demonstrate the degree of colonization of these organs by the GFP-marked clones. Fluorescent images in the left and middle columns represent projections of multiple sections and the images in the right column show a single plane. The discs and brains shown are representative of several samples. All images were taken at the same magnification (x10) and the 200 μm scale bar in the bottom right applies to all the pictures. The females in all crosses were of the genotype *eyFLP;Act5C*,*GAL4*,*UAS-GFP;FRT82B*,*tubGal80* and the males were, from top to bottom, *UAS-RFP-attP40; FRT82B*, *UAS-RFP-attP2 (WT*, *RFP)*, *UAS-RFP-attP40; FRT82B*, *UAS-E4orf4-17*.*22 (WT*, *E4orf4)*, *UAS-RFP attP40; FRT82B*, *scribble*^*1*^*/TM6B (scrib*^*−*^, *RFP)*, *UAS-RFP-attP40; FRT82B*, *UAS-E4orf4-17*.*22, scribble*^*1*^*/TM6B (scrib*^*−*^ *E4orf4)*, *UAS-Ras*^*V12*^, *UAS-RFP-attP40; FRT82B (Ras*^*V12*^, *RFP)*, *UAS-Ras*^*V12*^*; FRT82B*, *UAS-E4orf4-17*.*22 (Ras*^*V12*^, *E4orf4)*, *UAS-Ras*^*V12*^, *UAS-RFP-attP40; FRT82B*, *scribble*^*1*^*/TM6B (Ras*^*V12*^, *scrib*^*−*^, *RFP)*, *UAS-Ras*^*V12*^*; FRT82B*, *UAS-E4orf4-17*.*22*, *scribble*^*1*^*/TM6B (RasV*^*12*^, *scrib*^*−*^, *E4orf4)*. **b** The percentage of adult eclosion, signifying fly survival, was determined for flies carrying clones of the indicated genotypes, expressing E4orf4 (E4orf4:+) or a control *RFP* gene (E4orf4: −) as described in **a**. *N* > 300, collected from a minimum of three independent experiments. Error bars represent standard deviation. **c** Representative eyes harboring clones of the indicated genotypes are shown. The skull symbols represent pupal-lethality. The 50 μm scale bar in the bottom right applies to all the pictures

As shown in Fig. 2a, eye discs and brains of 3rd instar larvae containing *WT* or *scrib*^*−*^ clones were similar in size and morphology, and E4orf4 expression did not greatly affect organ size, although it reduced clone sizes in the *scrib*^*−*^ background. Brains and eye discs of some larvae with *Ras*^*V12*^-overexpressing clones were slightly enlarged, but the alterations were not significant in comparison with larvae carrying *Ras*^*V12*^, E4orf4-coexpressing clones. There were also no dramatic changes in the overall organ morphology. In contrast, *Ras*^*V12*^-overexpression in combination with *scrib* deletion resulted in massive growth of brain and eye discs and loss of recognizable morphology, creating a huge tumor mass. Furthermore, whereas the GFP-marked mitotic clones with other genotypes were restricted to parts of the eye discs and to limited areas in the brain hemispheres, the *Ras*^*V12*^, *scrib*^*−*^ mitotic clones colonized the whole eye disc and brain area and invaded the ventral nerve cord (VNC), as described previously^26,30^. E4orf4 expression had a remarkable effect on these highly aggressive tumors, resulting in complete restoration of normal size and morphology of both eye discs and brains, as well as nearly complete elimination of GFP-marked clones, reducing their size to that of *WT* clones.

Next, we determined how generation of the mitotic clones, with or without E4orf4, affected fly viability at 24 ^o^C. As seen in Fig. 2b, 100% of flies with *WT* mitotic clones were viable, and E4orf4 expression reduced viability in the *WT* background by only 3%. The generation of *scrib*^*−*^ homozygous clones reduced fly viability by 12% in the absence of E4orf4, but the flies were 96.5% viable when E4orf4 was expressed in these clones. Clonal *Ras*^*V12*^ overexpression led to a complete loss of fly viability in both *WT* and *scrib*^*−*^ backgrounds. However, E4orf4 expression could rescue 97% of flies with *Ras*^*V12*^-expressing clones and 80% of flies with *Ras*^*V12*^, *scrib*^*−*^ mitotic clones.

The eye phenotypes of adult flies that emerged from this experiment demonstrated an improvement in eye morphology in flies with *scrib*^*−*^ mitotic clones expressing E4orf4, and similar eye morphologies were evident in flies expressing E4orf4 in *Ras*^*V12*^ and *Ras*^*V12*^, *scrib*^*−*^ mitotic clones (Fig. 2c).

Altogether, the results shown in Fig. 2 indicate that E4orf4 is highly potent in preventing the development of even very aggressive tumors, and consequently reduces the ensuing lethality and eye deformities caused by the tumors.

**Fig. 3:**
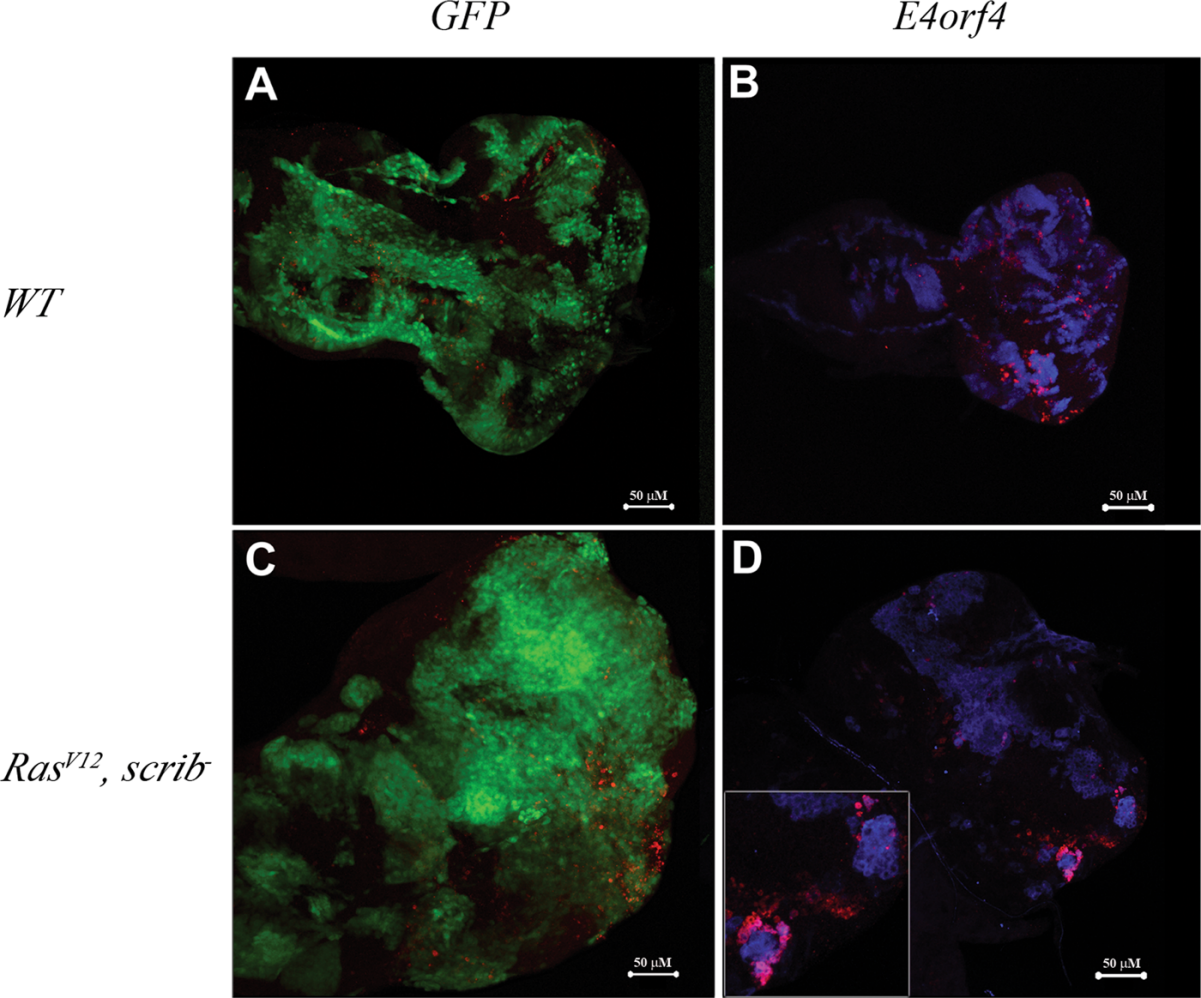
E4orf4-induced cell-death in *WT* and cancer clones. Third-instar larvae were collected at 24 ^o^C from crosses between *eyFLP;Act5C*,*GAL4*,*UAS-GFP;FRT82B*,*tubGal80* females and males of the following genotypes: *FRT82B*, *UAS-GFP* (**a**), *FRT82B*, *UAS-E4orf4-17*.*22* (**b**), *UAS-Ras*^*V12*^*; FRT82B*, *scrib*^*−*^, *UAS-GFP* (**c**), *UAS-Ras*^*V12*^*; FRT82B*, *scrib*^*−*^, *UAS-E4orf4-17*.*22* (**d**). Eye-antennal discs were dissected from these larvae and analyzed by confocal microscopy. GFP-expressing clones were visualized by GFP fluorescence and clones expressing E4orf4 were stained with E4orf4-specific antibodies (blue). Active (cleaved) Dcp1 staining is shown in red. All images represent projections of multiple sections. A representative eye disc is shown in each picture with a 50 μm scale bar. An enlarged inset of E4orf4-expressing *Ras*^*V12*^, *scrib*^*−*^ clones surrounded by concentrated active Dcp1 staining is displayed in **d**

As E4orf4 was reported to induce cell-death in flies and mammalian cells^12,13^, we examined whether cancer clone elimination could potentially result from E4orf4-induced cell-death manifested by activation of the Dcp1 caspase. Dispersed caspase activation, which was not concentrated near the strongest GFP-marked clones, was evident in control *WT* clones, as well as in *Ras*^*V12*^, *scrib*^*−*^ clones (Fig. 3a, c). Dcp1 activation was observed around strong E4orf4-expressing clones in the *WT* background (Fig. 3b), but the staining was somewhat dispersed in comparison to a robust accumulation of Dcp1 staining around *Ras*^*V12*^, *scrib*^*−*^ clones with strong E4orf4 expression (Fig. 3d). It should be noted that most of the E4orf4-expressing *Ras*^*V12*^, *scrib*^*−*^ clones have already been eliminated by this stage (Fig. 2a) and therefore it is likely that only remnants of the E4orf4-induced cell-death are still evident. Nonetheless, the results suggest that cell-death could explain, at least in part, the elimination of the large *Ras*^*V12*^, *scrib*^*−*^ tumor clones.

**Fig. 4:**
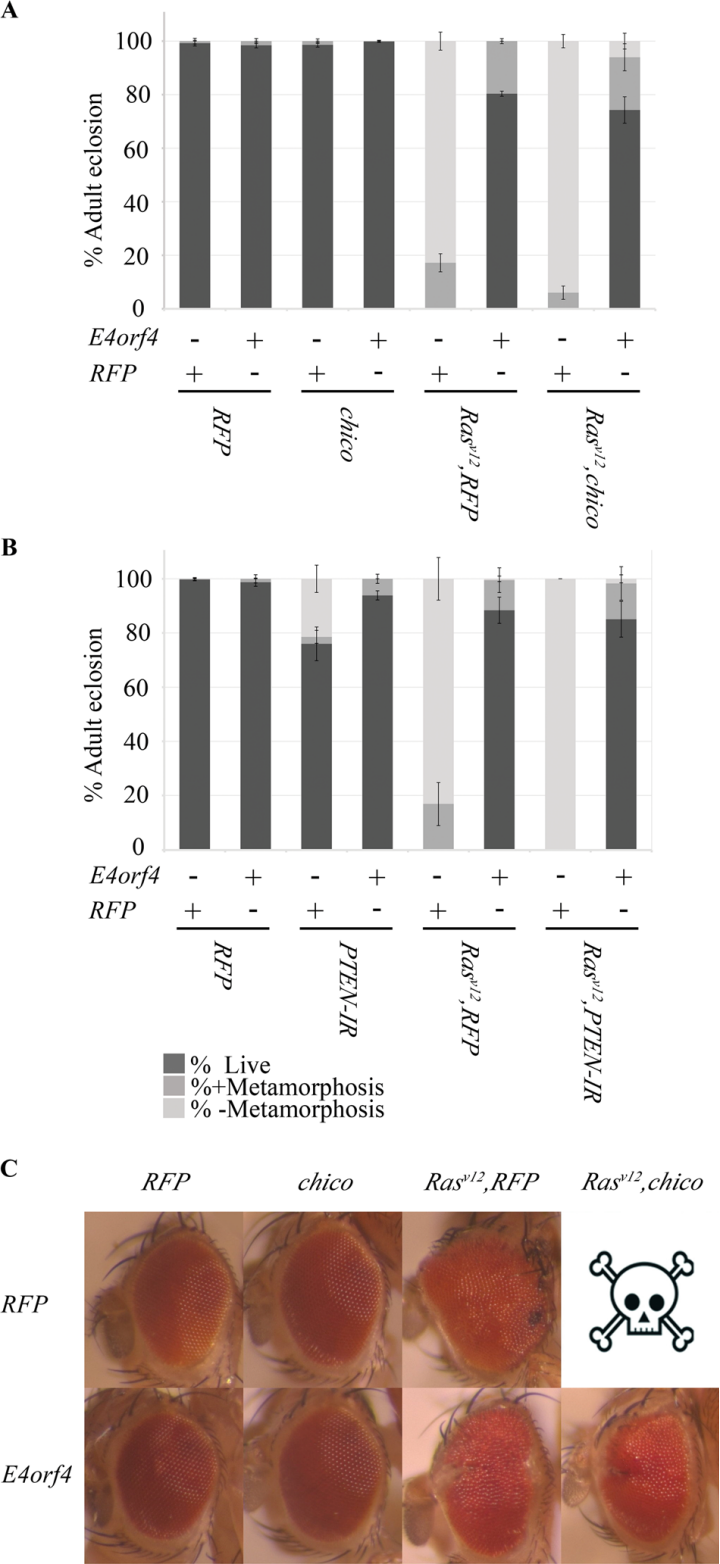
E4orf4 inhibits tumorigenesis induced by a combination of *Ras*^*V12*^ and activation of insulin-PI3K signaling. **a** The percentage of adult eclosion was determined for flies expressing RFP, or *chico*, or *Ras*^*V12*^ and RFP, or *Ras*^*V12*^ and *chico*, together with E4orf4 or *RFP*. The females in all crosses were of the genotype *ey-GAL4* and the males were, from left to right, RFP RFP: *UAS-RFP-attP40; UAS-RFP-attP2;* E4orf4 RFP: *UAS-E4orf4-attP40; UAS-RFP-attP2;* RFP *chico*: *UAS-RFP- attP40; UAS-chico;* E4orf4 *chico*: *UAS-E4orf4-attP40; UAS-chico;* RFP *Ras*^*V12*^ RFP: *UAS-Ras*^*V12*^, *UAS-RFP-attP40; UAS-RFP-attP2;* E4orf4 *Ras*^*V12*^ RFP: *UAS-Ras*^*V12*^, *UAS-E4orf4-attP40; UAS-RFP-attP2;* RFP *Ras*^*V12*^ *chico*: *UAS-Ras*^*V12*^, *UAS-RFP-attP40; UAS-chico*; E4orf4 *Ras*^*V12*^ *chico*: *UAS-Ras*^*V12*^, *UAS-E4orf4-attP40; UAS-chico*. All crosses were maintained at 24 ^o^C. Non-eclosed pupae were divided into two categories: those that have undergone metamorphosis and those that have not. *N* > 300, collected from three independent experiments. Error bars represent standard deviation. **b** The percentage of adult eclosion was determined for flies expressing RFP, or *PTEN-directed RNAi construct (PTEN-IR)*, or *Ras*^*V12*^ and RFP, or *Ras*^*V12*^ and *PTEN-IR*, together with E4orf4 or *RFP*. The females in all crosses were of the genotype *ey-GAL4* and the males were of the following genotypes: RFP RFP: *UAS-RFP- attP40; UAS-RFP-attP2;* E4orf4 RFP: *UAS-RFP- attP40; UAS-E4orf4-17*.*22;* RFP *PTEN-IR: UAS-PTEN-IR; UAS- RFP-attP2*; E4orf4 *PTEN-IR: UAS-PTEN-IR*; *UAS-E4orf4-17*.*22;* RFP *Ras*^*V12*^ RFP: *UAS-Ras*^*V12*^, *UAS-RFP-attP40; UAS-RFP-attP2;* E4orf4 *Ras*^*V12*^ RFP: *UAS-Ras*^*V12*^, *UAS-RFP-attP40; UAS-E4orf4-17*.*22;* RFP *Ras*^*V12*^ *PTEN-IR*: *UAS-Ras*^*V12*^, *UAS-PTEN-IR; UAS- RFP-attP2;* E4orf4 *Ras*^*V12*^ *PTEN-IR*: *UAS-Ras*^*V12*^, *UAS-PTEN-IR; UAS-E4orf4-17*.*22*. *N* > 300, collected from three independent experiments. Error bars represent standard deviation. **c** Representative eyes of flies obtained as described in **a** are shown. The pictures were captured at a magnification of x5. The skull symbol represents pupal-lethality and the absence of pupae that have undergone metamorphosis

In addition to the highly tumorigenic collaboration between activated *Ras*^*V12*^ and deletion of cell polarity-regulating genes, a collaboration between *Ras*^*V12*^ and activation of the insulin-PI3K pathway induces significantly more tumorigenesis than *Ras*^*V12*^ alone, possibly due to increased metabolism that supports cancer cell proliferation^27,31^. Activation of the insulin-PI3K pathway in *Drosophila* can be achieved by overexpression of Chico, the fly insulin receptor substrate, or by reduced expression of PTEN, the PI3K pathway inhibitor. To determine how E4orf4 affects PI3K-regulated tumorigenesis, E4orf4 or a control *RFP* transgene were expressed under *ey-GAL4* regulation together with various combinations of *Ras*^*V12*^, *chico*, and *PTEN* interfering RNA (*IR*), and fly viability was assayed. To detect differences between the effects of *Ras*^*V12*^ alone and *Ras*^*V12*^ supplemented by activation of PI3K, we counted separately empty pupal cases from which adult flies have eclosed, pupae containing pharate adults that failed to eclose, and pupae in which no metamorphosis took place. Figure 4a demonstrates that *chico* overexpression with or without concomitant expression of E4orf4 did not reduce significantly fly viability. Co-expression of *Ras*^*V12*^ with two copies of the *RFP* transgene caused 100% lethality at 24 ^o^C, whereas *Ras*^*V12*^, E4orf4 co-expression facilitated adult eclosion from 80% of the pupae. Co-expression of *chico* with *Ras*^*V12*^ aggravated the effect of *Ras*^*V12*^, increasing the percent of non-metamorphosing pupae from 83% to 94% (*p* = 0.005). However, even in this background, E4orf4 expression dramatically increased adult eclosion up to 74%.

Similar results were obtained when activation of the PI3K pathway was induced by knockdown of *PTEN* (*PTEN-IR*, Fig. 4b). Whereas *PTEN-IR* alone reduced fly viability to 76%, co-expression of E4orf4 rescued viability to 94% (*p* = 0.01). The concomitant expression of *PTEN-IR* aggravated the effect of *Ras*^*V12*^, increasing the percentage of non-metamorphosing pupae from 83% to 100% (*p* = 0.002). Here too, co-expression of E4orf4 resulted in a dramatic increase in adult eclosion to 85% in this background, very similar to a rescue to 88% eclosion in flies expressing *ey* *>* *Ras*^*V12*^ without *PTEN-IR*.

To investigate the effect of E4orf4 on tumor tissues, adult eyes were examined. As shown in Fig. 4c, the phenotype of *Ras*^*V12*^, E4orf4-expressing eyes was milder than that of eyes expressing *Ras*^*V12*^ alone, which were visualized in 0.6% adults that have eclosed. The ability of E4orf4 to rescue the eye phenotype of *Ras*^*V12*^, *chico* expressing flies could not be evaluated due to pupal-lethality of the *Ras*^*V12*^, *chico* genotype. As previously reported, E4orf4 expression caused a minor rough eye phenotype in a *WT* or *chico* backgrounds.

The results summarized in Fig. 4 confirm that E4orf4 is a potent inhibitor of tumorigenesis.

### E4orf4 partners that contribute to cancer elimination by E4orf4

**Fig. 5:**
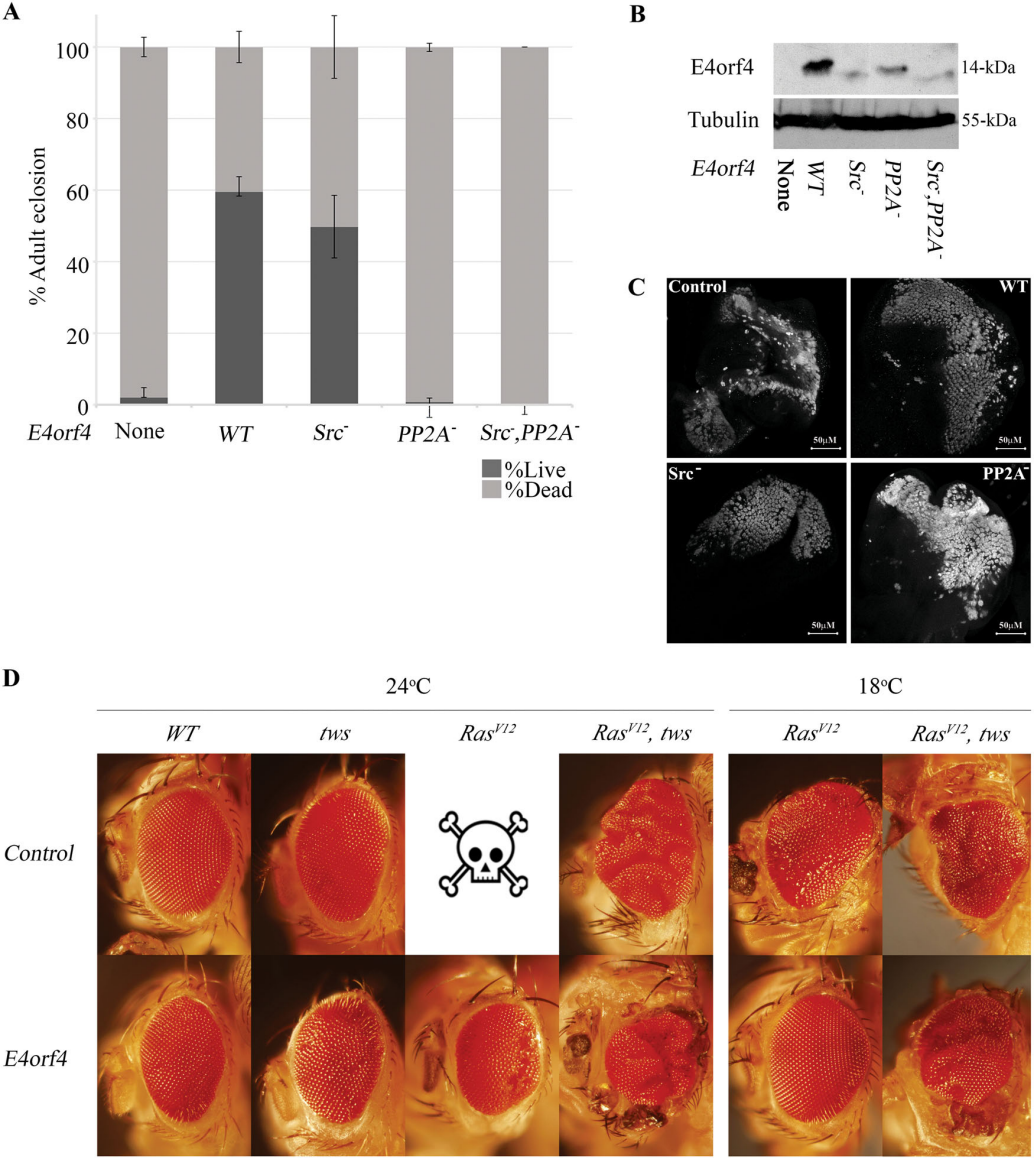
The contribution of the E4orf4 partners PP2A and Src to inhibition of *Ras*^*V12*^-induced tumorigenesis and toxicity by E4orf4. **a** Percentage of adult eclosion and non-eclosed pupae was determined in flies expressing *ey*-*GAL4* > *Ras*^*V12*^ together with control *RFP* (marked as E4orf4-None: *UAS-RFP-attP40; UAS-RFP-attP2*) or *WT* E4orf4 (*UAS-RFP-attP40; UAS-E4orf4-attP2)*, or the E4orf4 mutants that do not bind Src (Src^*−*^: *UAS-RFP-attP40; UAS-E4orf4* ^*R73/74/75A*^*-attP2*), or PP2A (PP2A^*−*^: *UAS-RFP- attP40; UAS-E4orf4* ^*R81F84A*^ *-attP2*, or the double mutant that does not bind both Src and PP2A (Src^*−*^, PP2A^*−*^: *UAS-RFP-attP40; UAS-E4orf4* ^*R73/74/75A +R81F84A*^*-attP2*). **b** Protein extracts were prepared from ten 3rd instar larvae expressing *RFP*, *WT* E4orf4, or the E4orf4 mutants described in **a**, using the *ey* driver at 24 ^o^C. A representative western blot is shown, stained with antibodies to E4orf4 and to Tubulin, which served as a loading control. **c** Eye-antennal imaginal discs obtained from crosses as described in **a** were dissected from age-matched 3rd-instar larvae grown at 24 ^o^C. The discs were stained with antibodies to ELAV and were analyzed by confocal microscopy. All images were taken at the same magnification (x40) and represent projections of multiple sections. A representative eye disc is shown in each picture with a 50 μm scale bar. **d** Representative eyes of flies harboring clones of the indicated genotypes and reared at 18 ^o^C or 24 ^o^C are shown. Pictures were captured at a magnification of x5. A skull symbol represents pupal-lethality. The females in all crosses were of the genotype *eyFLP;Act5C*,*GAL4*,*UAS-GFP;FRT82B*,*tubGal80* and the males were, in the upper row *WT*: *FRT82B*,*UAS-GFP*, *tws: FRT82B*,*tws*^*60*^*/TM6B*, *Ras*^*V12*^*: UAS-Ras*^*V12*^*;FRT82B*,*UAS-GFP*, *Ras*^*V12*^, *tws: UAS-Ras*^*V12*^*;FRT82B*,*tws*^*60*^*/TM6B* and in the lower panel, *WT*: *FRT82B*,*UAS-E4orf4-17*.*22*, *tws: FRT82B*,*tws*^*60*^,*UAS-E4orf4-17*.*22/TM6B*, *Ras*^*V12*^*:UAS-Ras*^*V12*^*;FRT82B*,*UAS-E4orf4-17*.*22*, *Ras*^*V12*^, *tws: UAS-Ras*^*V12*^*;FRT82B*,*tws*^*60*^,*UAS-E4orf4-17*.*22/TM6B*. It should be noted that even though flies with the *tws* mutation did not express a control GFP transgene to match the E4orf4 transgene whereas the other flies contained this control, potential competition for the *GAL4* driver by *UAS-E4orf4* did not lead to rescue of eye morphology in the mutant background

We previously reported that two E4orf4 partners, PP2A and Src, contributed in an additive manner to the mild E4orf4-induced cell-death in normal *Drosophila* tissues^12^. We now tested whether these partners contributed similarly to the E4orf4-induced elimination of cancer tissues. The flies used in this experiment carried a *UAS-Ras*^*V12*^ transgene on the 2nd chromosome and an additional transgene integrated in the *attP2* site on the 3rd chromosome containing a *UAS*-driven cDNA of *RFP*, *WT* E4orf4, or E4orf4 mutants that cannot bind Src (“Src^*−*^”) or PP2A (“PP2A^*−*^”), or both (“Src^*−*^, PP2A^*−*^”)^12^. Integration of all transgenes at the same chromosomal site minimizes position effects on their expression. Flies of these genotypes were crossed to *ey* *>* *GAL4* flies at 24 ^o^C. As shown in Fig. 5a, *ey-GAL4*-driven *Ras*^*V12*^ expression in the presence of a control *RFP* gene reduced fly viability and only 2% of the flies reached adulthood. Despite the relatively low expression levels of *WT* E4orf4 integrated at the *attP2* site (Fig. 1c), E4orf4 was still able to increase viability of *ey* *>* *Ras*^*V12*^-expressing flies to 60%. The Src^*−*^ E4orf4 mutant that is unable to bind Src kinase was only slightly less efficient than *WT* E4orf4 in rescuing the viability of *ey* *>* *Ras*^*V12*^-expressing flies, leading to 50% adult eclosion. In contrast, the PP2A^*−*^ E4orf4 mutant, which could not bind PP2A, lost completely the ability to counteract *Ras*^*V12*^ activity, and fly viability remained 0.7%. Although the *WT* E4orf4 protein accumulated at higher levels than the E4orf4 mutants, the dissimilarity in function of the two mutants cannot be explained by differences in protein levels, as the levels of the PP2A^*−*^ mutant were consistently higher than the levels of the Src^*−*^ mutant (Fig. 5b). The double mutant, PP2A^*−*^ Src^*−*^, which lost the ability to bind both Src and PP2A was also unable to increase fly viability, resulting in 0% adult eclosion.

In addition to examining the ability of E4orf4 mutants to rescue the viability of *ey* *>* *Ras*^*V12*^-expressing flies, we evaluated their ability to rescue eye disc differentiation. Eye discs of 3rd instar larvae that expressed *Ras*^*V12*^ together with RFP or E4orf4 under *ey* *>* *GAL4* regulation were stained with anti-ELAV antibodies. As seen before (Fig. 1a), expression of *Ras*^*V12*^ in the absence of E4orf4 resulted in loss of normal differentiation marked by irregular ELAV staining (Fig. 5c). WT E4orf4 expression (the “weak1” transgene) enhanced normal differentiation, as did expression of the Src^*−*^E4orf4 mutant. In contrast, the PP2A^*−*^ E4orf4 mutant was much less efficient in recovering normal ommatidia patterns, further confirming its reduced ability to counteract *Ras*^*V12*^ effects.

To validate the contribution of PP2A to the ability of E4orf4 to counteract *Ras*^*V12*^ activities, the influence of E4orf4 on the *Ras*^*V12*^ induced eye phenotype was compared in *WT* eyes and in eyes lacking *tws*, the fly PP2A-B55 subunit that mediates the E4orf4-PP2A interaction^13,14^. Figure 5d demonstrates that E4orf4 caused a minor rough eye phenotype in both *WT* and *tws* backgrounds, possibly through Src binding. *Ras*^*V12*^ expression at 24 ^o^C was lethal and no adult progeny eclosed. Concomitant expression of E4orf4 rescued fly viability and produced eyes with some defects, which retained regular differentiation in large areas. In contrast, the *tws* mutation reduced *Ras*^*V12*^-induced lethality and adult eyes with a typical “raspberry-like” appearance were observed. However, E4orf4 expression did not improve eye morphology in this background. To overcome the *Ras*^*V12*^-induced lethality, flies were additionally reared at 18 ^o^C. At this temperature, ey > *Ras*^*V12*^ flies could eclose and they presented rough, overproliferating eye tissues and some small growths, whereas E4orf4 expression reverted eye morphology to near normal. In the *tws* background, the ey > *Ras*^*V12*^ eyes remained abnormal upon E4orf4 expression.

The findings shown in Fig. 5 demonstrate that the interaction with PP2A is crucial for the ability of E4orf4 to rescue flies from *Ras*^*V12*^–induced tumorigenesis and lethality, but surprisingly, the interaction of E4orf4 with Src has only a minor contribution to counteracting *Ras*^*V12*^-induced lethality. This differs from the situation in normal fly tissues, in which PP2A and Src contribute additively to E4orf4 function^12^.

### E4orf4 rescues effects of early *scrib* elimination even when expressed at a late stage

**Fig. 6:**
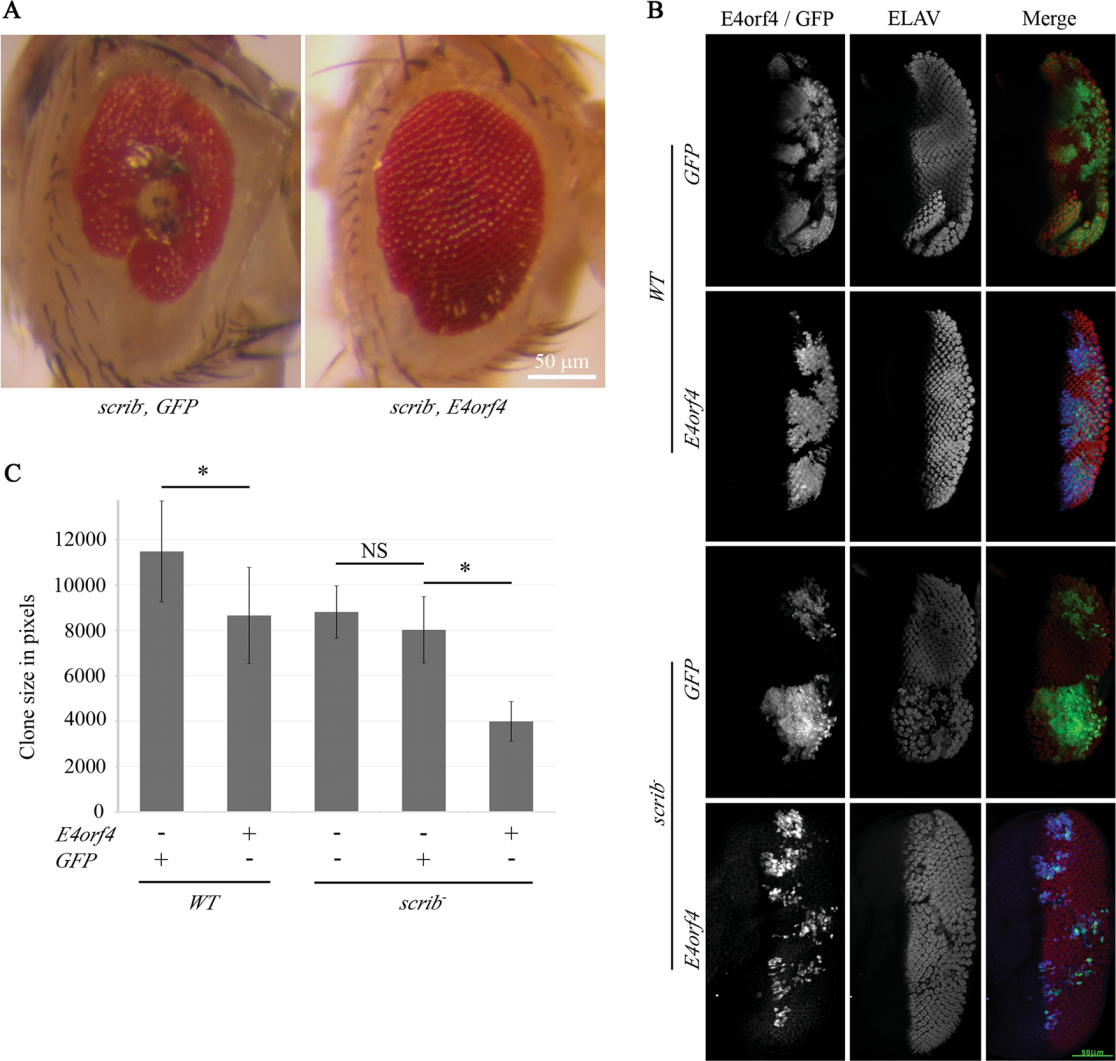
Late expression of E4orf4 rescues effects of early *scrib* deletion. **a** Representative eyes carrying homozygous *scrib* mutant clones induced by *ey* *>* *FLP* and expressing either *GFP* or *WT* E4orf4 under the regulation of *GMR-GAL4* are shown. Flies of the genotype *yw*,*eyFLP;19A*,*UAS-med8GFP2A;FRT82B*,*tubGAL80/TM3* were crossed to flies of the following genotypes: *GMR-GAL4; FRT82B*, *UAS-GFP*, *scribble*^*1*^*/TM6B (scrib*^*−*^, *GFP)*, *GMR-GAL4; FRT82B*, *UAS-E4orf4-17*.*22*, *scribble*^*1*^*/TM6B (scrib*^*−*^, *E4orf4)*. The 50 μm scale bar in the bottom right applies to both pictures. **b** Eye-antennal discs were dissected from 3rd-instar larvae grown at 24 ^o^C and analyzed by confocal microscopy. Flies with *scrib*^*−*^ clones were as described in **a**. Flies with *WT* clones were generated by a cross between *yw*,*eyFLP;19A*,*UAS-med8GFP2A;FRT82B*,*tubGAL80/TM3* and *GMR-GAL4; FRT82B*,*UAS-GFP (WT*, *GFP) or GMR-GAL4; FRT82B*, *UAS-E4orf4-17*.*22 (WT*, *E4orf4)*. All images were taken at the same magnification (x40) and represent projections of multiple sections. *WT* and *scrib*^*−*^ clones, as indicated in the figure, are marked by GFP fluorescence (green) or stained with antibodies to E4orf4 (blue) (left column). The discs were also stained with ELAV-specific antibodies (red)(middle column) to visualize their state of differentiation. A merge of all stains is seen in the right column. A representative eye disc is shown in each picture. The 50 μm scale bar in the bottom right applies to all the pictures. **c** The sizes of GFP-marked clones from eye-antennal discs with *WT* or *scrib*^*−*^ mutant backgrounds were detected by GFP fluorescence (present also in E4orf4-expressing clones, but not shown in **b**) and quantified using ImageJ software. Average pixel numbers are shown. All the discs used here were age-matched. *N* > 8 from > 3 independent experiments, error bars represent the standard deviation. Statistical significance was determined by a student *t*-test. *p < 0.05, NS: not significant

In the previous experiments, the concomitant expression of *Ras*^*V12*^ and E4orf4 was induced by the *ey* driver (Figs. 1, 4, and 5), or was induced following *ey* *>* *FLP* activation together with *scrib* deletion (Fig. 2). Thus, the results of these experiments show that E4orf4 inhibits tumorigenesis when expressed simultaneously with cancer induction. To examine the consequences of late E4orf4 expression, *scrib*^*−*^ GFP-marked homozygous clones were induced under the regulation of ey > *FLP*, which is expressed in the eye disc as early as the end of the 1st instar larval stage^33^. In contrast, the expression of E4orf4 or a *GFP* control gene was driven by *GMR*-*GAL4*, which is activated in the eye disc only at the 3rd instar larval stage^34^. As seen in Fig. 6a, the eyes of flies with mitotic clones lacking *scrib* and expressing the control *GFP* gene were abnormal, with small growths and necroses. However, *GMR* *>* *E4orf4* expression resulted in a significant suppression of this adult eye phenotype. We have previously shown that, in *WT* background, *GMR* *>* *E4orf4* on its own induced only a mild rough eye phenotype^12^.

To evaluate the size and state of differentiation of *scrib*-deficient clones, the eye discs of 3rd instar larvae containing GFP-marked mitotic clones were co-stained for ELAV and E4orf4. As seen in Fig. 6b, GFP-marked *scrib*-deficient clones were relatively large and ELAV staining in these clones was irregular compared with *WT* clones, indicating abnormal differentiation. In contrast, *scrib*-deficient clones that expressed E4orf4 were smaller and the pattern of ELAV staining resembled more closely the staining in *WT* clones, although E4orf4 had some effect on photoreceptor differentiation as the spacing between the ommatidia appeared increased. It should be noted that because the expression of E4orf4 and GFP was driven by *GMR-GAL4*, it was seen only in the *ey* *>* *FLP-* generated clones, in which the *GAL4* system inhibitor Gal80 was removed by recombination. Figure 6c demonstrates that whereas E4orf4 reduced the size of *WT* clones to 72%, it reduced the size of *scrib*-deficient clones to 43% compared to the GFP-expressing control. Statistical analysis (Summary Independent-Samples Test) further demonstrated that the decrease in cancer clone sizes induced by E4orf4 was significantly larger than the E4orf4-induced decrease in *WT* clone sizes (*p* = 0.012). These findings indicate that E4orf4 can specifically eliminate *scrib*^*−*^ clones and rescue adult eye morphology even when expressed at a much later stage than *scrib* elimination, and that its toxic effects on *WT* cells are much milder than its toxic effects in a cancer tissue.

## Discussion

### E4orf4 effects in various types of tumors

Previous work in tissue culture revealed that E4orf4-induced cell-death was more efficient in mammalian cancer cells than in normal cells^13^. By utilizing the power of fly genetics, the present work demonstrates that E4orf4 can also strongly suppress the tumorigenic process in a whole organism with minimal damage to normal tissues.

Various types of tumors were used in this study: Hyperplastic *Ras*^*V12*^-induced tumors, which over-proliferate and cause imaginal disc overgrowth without metastasizing; Neoplastic *scrib*^*−*^ tumors, which lose the monolayered organization and correct apical-basal polarity of wild-type epithelia^35^ and are deficient in normal differentiation as well as prone to metastasis; and highly aggressive and metastatic *Ras*^*V12*^, *scrib*^*−*^ tumors. In addition, we examined the effects of E4orf4 on tumors induced by combined activation of the Ras and insulin-PI3K pathways. The tumor types investigated here are highly relevant to human tumors. Activation of *Ras* signaling occurs in ~30% of human cancers^36^ and deregulation of *scrib*, as well as of many other cell polarity-regulating genes contributes to many types of human tumors^37^. Disruption of insulin-PI3K signaling is also quite common in human cancers^38^.

This work shows that E4orf4 was efficient in inhibiting tumorigenesis in representatives of all types of tumor tissues described above. Especially exciting was the ability of E4orf4 to prevent formation of the massive metastatic tumors induced by the combination of *Ras*^*V12*^ overexpression and *scrib* deletion (Fig. 2). E4orf4 succeeded in preventing both overgrowth and the ability of cancer cells to totally colonize the tissue and invade the VNC. The mild effects of E4orf4 on normal tissues were reaffirmed.

E4orf4 induction of cell-death is responsible, at least in part, for its anti-tumorigenic effect. Although technical difficulties prevented the visualization of E4orf4-induced cell-death in early 2nd instar larvae shortly after initiation of E4orf4 expression in the highly aggressive *Ras*^*V12*^, *scrib*^*−*^ tumors, Dcp1 activation was evident around clones with strong E4orf4 expression in 3rd instar larvae (Fig. 3). This observation suggests that induction of cell-death played a role in the elimination of tumorous clones. E4orf4 is also known to kill normal cells, but to a lesser degree^12^, and thus the elimination of aggressive *Ras*^*V12*^, *scrib*^*−*^ cancer clones as well as of *scrib*^*−*^ clones was more efficient than removal of *WT* clones (Figs. 2a and 6c). Effects on cell proliferation could also potentially contribute to cancer elimination by E4orf4 but we found no evidence supporting this possibility.

The ability of E4orf4 to revert the effects of *scrib* deletion when expressed at a later stage (Fig. 6) is also interesting. *scrib*^*−*^ clones were shown to be eliminated by their *WT* neighbors and by JNK-dependent apoptosis, causing eye deformities^30,39,40^. It is therefore possible that E4orf4, which inhibits classical apoptosis in normal cells^12^, could also inhibit apoptosis in the *scrib*^*−*^ clones, thus rescuing normal eye morphology. It is also possible, however, that E4orf4 reverted the neoplastic identity of these clones through other mechanisms.

### The contribution of E4orf4 partners

Two E4orf4 partners, PP2A and Src kinase contribute additively to E4orf4-induced cell-death both in mammalian transformed cell lines^32^ and in normal *Drosophila* tissues^12^. The major surprising finding here was that PP2A binding was crucial for the ability of E4orf4 to inhibit Ras carcinogenesis in vivo, whereas Src binding played a marginal role (Fig. 5). Several types of PP2A holoenzymes act as tumor suppressors, and mutations in the scaffolding A subunit, as well as in some regulatory B subunits, were found in various types of human cancer^41^. Moreover, PP2A activation by small molecules was shown to inhibit growth of some tumors^42,43^. In contrast, in some cellular contexts the PP2A B55 subunit was reported to promote tumorigenesis^44^ and certain PP2A inhibitors are tested as chemotherapeutics^43^. Thus, PP2A activity must be carefully balanced in the cellular context. The mechanisms by which PP2A supports E4orf4-induced cell-death are still debated. Although one report suggested that inhibition of PP2A activity contributed to this process^45^, several findings indicate that the E4orf4-PP2A complex possesses PP2A phosphatase activity^14^ and that PP2A contributes positively to E4orf4-induced cell-death^13,15^. Furthermore, PP2A is recruited by E4orf4 to several substrates involved in its functions, including induction of cell-death^2,46^. Thus, recruitment of PP2A to novel substrates or stabilization of existing interactions may shift the balance in several E4orf4-targeted cellular pathways. Cancer cells may be more sensitive to these changes than normal cells, leading to cell-death. As one example, E4orf4 inhibits some branches of the DDR in a PP2A-dependent manner^3^. Many cancer cells have deficiencies in DNA damage signaling and may rely more heavily on their remaining intact DDR pathways. Targeting the remaining pathways by a DDR inhibitor such as E4orf4 may therefore be selectively toxic to these cancer cells.

### The potential of E4orf4 for cancer therapy

This work demonstrated the remarkable activity of E4orf4 as an anticancer agent in vivo, providing a strong incentive for development of E4orf4-based cancer treatments. The potential advantages of E4orf4-based cancer therapy include the following: (1) E4orf4 kills several types of tumor cell lines but not normal cells^6,8,13,47,48^. (2) E4orf4 induces p53-independent cell-death^8,49^, which could be effective in p53-deficient tumors. (3) E4orf4 induces caspase-independent cell-death^5^, which could be useful in cancers with deficiencies in classical apoptosis. (4) As E4orf4 induces a unique mode of programmed cell-death by targeting a combination of several cellular pathways^2,3^, it may provide alternative solutions in cases of therapy-resistant disease. Therefore, design of E4orf4-based cancer therapies would be highly beneficial. Cell polarity genes as well as the PI3K pathway are associated with the formation of diverse types of cancer^23,24,38^, and providing appropriate therapy would be beneficial. Moreover, despite more than three decades of research, no anti-*Ras* therapies have become available^50^. Therefore, finding effective treatment of cancers with *Ras* activation^36^ would in itself be a satisfactory result of using E4orf4-based cancer therapy.

## Materials and methods

### Fly culture

Fly stocks (Table 1) were cultured on standard cornmeal/yeast media. Crosses were maintained at 18 °C, 24 °C, or 29 °C depending on the required experimental conditions. Each cross included ten virgin females and five males. Parents were allowed to mate for 3 days and were then transferred to a new vial for additional 3 days and were then discarded.

**Table 1:** Fly stocks

Described in FlyBase (http://flybase.org/):
*yw*,*eyFLP;19A*,*UAS-med8GFP2A;FRT82B*,*tubGAL80/TM3*
*ey-GAL4*
*GMR-GAL4*
The *PTEN-IR* (KK101475) stock was obtained from the Vienna *Drosophila* Resource Center (VDRC, Vienna, Austria). The following stocks were kindly provided to us*: yw*,*eyFLP;Act5C*,*GAL4*,*UAS-GFP;FRT82B*,*tubGal80* from T. Xu, *UAS-Ras*^*V12*^ from G. Halder, *scribble*^*1*^ from D. Bilder (called here *scrib*^*−*^), *UAS-chico* from H. Stocker and *FRT82B*, *tws*^*60*^*/TM6B* by T. Uemura (Kyoto University, Kyoto, Japan).
The following strains were generated in our laboratory and are described in ref. ^12^:
*UAS-RFP-attP40*, *UAS-RFP-attP2*, *UAS-E4orf4-17*.*22*, *UAS-E4orf4-attP40*, *UAS-E4orf4-attP2*, *UAS-E4orf4*^*R81F84A*^*-attP40* (“PP2A^*−*“^,^48^), *UAS-E4orf4*^*R73/74/75A*^*-attP40* (“Src^*−*^”,^12^), and *UAS-E4orf4*^*R81F84A*^ *+* ^*R73/74/75A*^*-attP40* (Src^*−*^, PP2A^*−*^)^12^.
The following strains were generated in our laboratory by meiotic recombination:
*FRT82B*, *UAS-GFP*
*FRT82B*, *UAS-E4orf4-17*.*22*
*FRT82B*, *tws*^*60*^, *UAS-E4orf4-17*.*22/TM6B*
*UAS-Ras*^*V12*^*; FRT82B*, *UAS-GFP*
*UAS-Ras*^*V12*^*; FRT82B*, *UAS-E4orf4-17*.*22*
*UAS-Ras*^*V12*^*; FRT82B*, *tws*^*60*^, *UAS-E4orf4-17*.*22/TM6B*
*UAS-RasV12; FRT82B*, *tws*^*60*^*/TM6B*
*UAS-RFP-attP40; FRT82B*, *UAS-RFP-attP2*
*UAS-RFP- attP40; FRT82B*, *UAS-E4orf4-17*.*22*
*UAS-RFP- attP40; FRT82B*, *scribble*^*1*^*/TM6B*
*UAS-RFP- attP40; FRT82B*, *UAS-E4orf4-17*.*22, scribble*^*1*^*/TM6B*
*UAS-RFP- attP40; FRT82B*
*UAS-Ras*^*V12*^, *UAS-RFP-attP40; FRT82B*
*UAS-Ras*^*V12*^, *UAS-RFP-attP40; FRT82B*, *scribble*^*1*^*/TM6B*
*UAS-Ras*^*V12*^*; FRT82B*, *UAS-E4orf4-17*.*22*, *scribble*^*1*^*/TM6B*
*UAS-RFP- attP40; UAS-RFP-attP2*
*UAS-E4orf4- attP40; UAS-RFP-attP2*
*UAS-RFP- attP40; UAS-chico*
*UAS-E4orf4- attP40; UAS-chico*
*UAS-Ras*^*V12*^, *UAS-RFP-attP40; UAS-RFP-attP2*
*UAS-Ras*^*V12*^, *UAS-E4orf4-attP40; UAS-RFP-attP2*
*UAS-Ras*^*V12*^, *UAS-RFP-attP40; UAS-chico*
*UAS-Ras*^*V12*^, *UAS-E4orf4-attP40; UAS-chico*
*PTEN-IR; UAS-E4orf4-17*.*22*
*PTEN-IR; UAS- RFP-attP2*
*UAS-Ras*^*V12*^, *PTEN-IR; UAS- RFP-attP2*
*UAS-Ras*^*V12*^, *UAS-RFP-attP40; UAS-E4orf4-17*.*22*
*UAS-Ras*^*V12*^, *PTEN-IR; UAS-E4orf4-17*.*22*
*UAS-RFP- attP40; UAS-E4orf4- attP2*
*UAS-RFP- attP40; UAS-E4orf4* ^*R81F84A*^ *- attP2*
*UAS-RFP- attP40; UAS-E4orf4* ^*R73/74/75A*^ *- attP2*
*UAS-RFP- attP40; UAS-E4orf4*^*R81F84A*^ *+* ^*R73/74/75A*^*- attP2*
*UAS-Ras* ^*V12*^ *; UAS-E4orf4-attP2*
*UAS-Ras* ^*V12*^ *; UAS-E4orf4* ^*R81F84A*^ *- attP2*
*UAS-Ras* ^*V12*^ *; UAS-E4orf4* ^*R73/74/75A*^ *- attP2*
*UAS-Ras*^*V12*^*; UAS-E4orf4*^*R81F84A*^ *+* ^*R73/74/75A*^*- attP2*
*GMR-GAL4; FRT82B*
*GMR-GAL4; FRT82B*, *UAS-E4orf4-17*.*22*
*GMR-GAL4; FRT82B*, *scribble*^*1*^*/TM6B*
*GMR-GAL4; FRT82B*, *UAS-GFP*, *scribble*^*1*^*/TM6B*
*GMR-GAL4; FRT82B*, *UAS-E4orf4-17*.*22*, *scribble*^*1*^*/TM6B*

### Adult eclosion test

The number of empty pupal cases from which adult flies have eclosed and of pupae that died before or after metamorphosis were counted in each vial for up to 3 weeks after pupa formation. Eclosed adults were removed daily. The ratio between empty pupal cases and the total number of pupae was calculated.

### Phenotypic characterization of adult eyes

Adult flies were anesthetized with CO2. Equal numbers of female eyes were examined for each genotype (*n* > 20 unless otherwise stated). The eyes were photographed using AxioCam MRc and Zeiss Discovery V8 binocular. To represent the depth of field in full, a series of images with different foci were taken, and the images were stacked using the Helicon Focus software.

### Western blots

Whole-cell extracts were prepared from 3rd-instar larvae in lysis buffer [50 mM Tris·HCl (pH 7.4), 250 mM NaCl, 5 mM EDTA, 0.1% Triton X-100, 0.5% Nonidet P-40, and a 1/10 volume of Complete protease inhibitor mixture (Roche, Basel, Switzerland)]. Proteins were analyzed by western blots using antibodies to E4orf4^8^ and alpha-Tubulin (Sigma, Rehovot, Israel).

### Staining imaginal discs

Larval tissues were dissected and stained following standard procedures. Primary antibodies used: mouse monoclonal anti-E4orf4 (#16, 1:100)^8^; rat monoclonal anti-ELAV (hybridoma bank, 9F8A9), rabbit activated (cleaved) Dcp1-specific antibody (Cell Signaling Technology, Danvers, MA, USA). Secondary fluorescent antibodies for fluorescent staining were Cy2, Cy3, or Cy5-conjugated anti-rabbit/mouse/rat (1:100) (Jackson Immunoresearch, West Grove, PA, USA). Samples were mounted with a DakoCytomation mounting medium (Agilent-Dako, Santa Clara, CA, USA).

### Clone size quantification

The size of GFP-positive clones was measured in pixels using the ImageJ software (NIH). Briefly, images of all samples were captured using the same magnification and acquisition conditions in a confocal microscope. A *Z*-stack was formed from all eye-antennal discs and a maximum intensity projection was created to include the entire depth of the discs. Eye disc size was quantified by increasing the brightness/contrast level marking the disc margins and measuring its area. The area of GFP-marked clones with intensities above a defined threshold were measured and summarized.

### Data analysis

The statistical significance of differences in eclosion rates or clone sizes between the various fly groups were calculated by an unpaired *t*-test. Summary Independent-Samples Test was used to assess whether the differences between E4orf4-induced changes in *WT* and *scrib*^*−*^ clone sizes were significant. This statistical analysis was carried out using the SPSS software package (Release 23.0.0.0, SPSS Inc., 2014).
